# Recent Advances in the Development of Toll-like Receptor Agonist-Based Vaccine Adjuvants for Infectious Diseases

**DOI:** 10.3390/pharmaceutics14020423

**Published:** 2022-02-16

**Authors:** Jing-Xing Yang, Jen-Chih Tseng, Guann-Yi Yu, Yunping Luo, Chi-Ying F. Huang, Yi-Ren Hong, Tsung-Hsien Chuang

**Affiliations:** 1 Immunology Research Center, National Health Research Institutes, Miaoli 35053, Taiwan; jingxingyang@nhri.edu.tw (J.-X.Y.); mark0918@nhri.edu.tw (J.-C.T.); 2 National Institute of Infectious Diseases and Vaccinology, National Health Research Institutes, Miaoli 35053, Taiwan; guannyiy@nhri.edu.tw; 3 Department of Immunology, Institute of Basic Medical Sciences, Chinese Academy of Medical Sciences, School of Basic Medicine, Peking Union Medical College, Beijing 100005, China; ypluo@ibms.pumc.edu.cn; 4 Institute of Biopharmaceutical Sciences, College of Pharmaceutical Sciences, National Yang Ming Chiao Tung University, Taipei 112304, Taiwan; cyhuang5@nycu.edu.tw; 5 Graduate Institute of Medicine, College of Medicine, Kaohsiung Medical University, Kaohsiung 80708, Taiwan; m835016@kmu.edu.tw; 6 Department of Life Sciences, National Central University, Taoyuan City 32001, Taiwan; 7 Program in Environmental and Occupational Medicine, Kaohsiung Medical University, Kaohsiung 80708, Taiwan

**Keywords:** adjuvant, nasal adjuvant, toll-like receptor, vaccine, mRNA vaccine

## Abstract

Vaccines are powerful tools for controlling microbial infections and preventing epidemic diseases. Efficient inactive, subunit, or viral-like particle vaccines usually rely on a safe and potent adjuvant to boost the immune response to the antigen. After a slow start, over the last decade there has been increased developments on adjuvants for human vaccines. The development of adjuvants has paralleled our increased understanding of the molecular mechanisms for the pattern recognition receptor (PRR)-mediated activation of immune responses. Toll-like receptors (TLRs) are a group of PRRs that recognize microbial pathogens to initiate a host’s response to infection. Activation of TLRs triggers potent and immediate innate immune responses, which leads to subsequent adaptive immune responses. Therefore, these TLRs are ideal targets for the development of effective adjuvants. To date, TLR agonists such as monophosphoryl lipid A (MPL) and CpG-1018 have been formulated in licensed vaccines for their adjuvant activity, and other TLR agonists are being developed for this purpose. The COVID-19 pandemic has also accelerated clinical research of vaccines containing TLR agonist-based adjuvants. In this paper, we reviewed the agonists for TLR activation and the molecular mechanisms associated with the adjuvants’ effects on TLR activation, emphasizing recent advances in the development of TLR agonist-based vaccine adjuvants for infectious diseases.

## 1. Introduction

**Figure 1:**
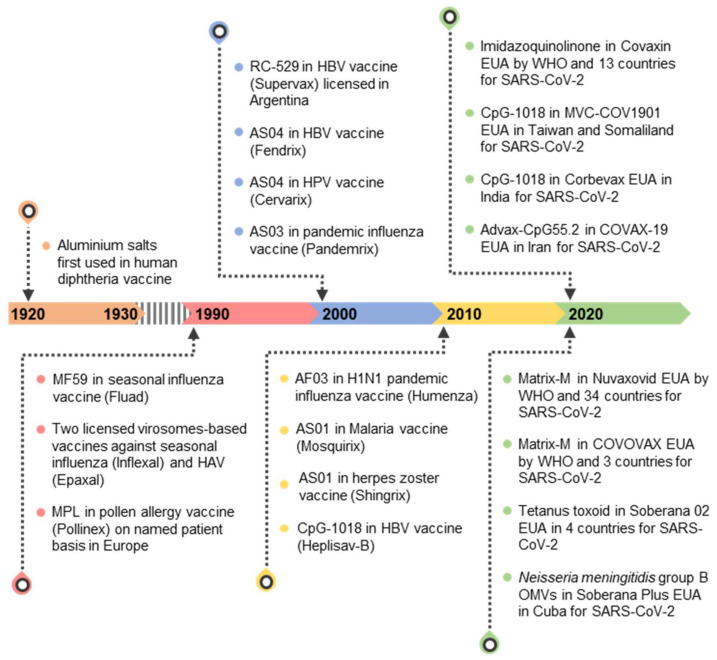
A timeline of adjuvant development and vaccine licensing. The year the adjuvant was used in licensed vaccines or emergency use authorized (EUA) vaccines is shown. AS, adjuvant system; MPL, monophosphoryl lipid A; HAV, hepatitis A virus; HBV, hepatitis B virus; HPV, human papillomavirus; WHO, world health organization; OMVs, outer membrane vesicles.

Since they were first introduced more than 200 years ago, vaccines have been used as powerful tools to reduce the morbidity and mortality of infectious diseases. Early vaccines were mainly generated by the inactivation or attenuation of live viruses, which stimulate immune responses without causing disease. Adjuvants have been used as parts of vaccines since the beginning of the 20th century when traditional vaccines failed to generate effective immune responses to protect the host against microbial infections [1,2,3]. In the 1920s, the discovery of the adjuvant effect of aluminum salts (alum) to boost the immune response to diphtheria and tetanus toxoids was a milestone. Alums have been used consistently in vaccines, and for several decades they were the only adjuvants used in vaccines [4,5]. In the late 1980s, recombinant DNA and protein expression technologies paved the way for the development of protein antigens to be used as subunit vaccines. The majority of recombinant antigens, however, showed weak immunogenicity even in the presence of alum. To overcome immunogenicity barriers, a new wave of development focused on using new adjuvants to increase the efficacy of vaccines. In the late 1990s, MF59 was formulated and first used as adjuvant in an approved influenza vaccine. Following the development of MF59, there have been several other adjuvants developed and employed in licensed human vaccines, including AS01, AS03, AF03, AS04, and CpG-1018 (Figure 1). These modern adjuvants have different functional mechanisms than the traditional alum adjuvant [6,7,8,9,10].

Alum, itself, can be classified into different types including aluminum hydroxide, aluminum phosphate, and amorphous aluminum hydroxyphosphate sulfate. Although these different types of alums have distinct physicochemical properties, their modes of action all involve absorption of the antigen in the formulated vaccine. Their similar modes of action result in what is known as the “depot effect,” in which the antigen is stably released at the injection site in order to prolong its exposure to immune cells and enhance immune responses [11,12]. MF59, AS03, and AF03 are all squalene-based emulsion adjuvants. MF59 contains squalene, polysorbate 80, sorbitan trioleate, and trisodium citrate dehydrate. AS03 contains squalene, polysorbate 80, and α-tocopherol. AF03 is composed of squalene, polyoxyethylene cetostearyl ether, mannitol, and sorbitan oleate. Squalene, the common component of these adjuvants, exists mainly in an oil phase in these oil-in-water emulsion adjuvants. Squalene is a natural lipid in the human body that is generated in the cholesterol synthesis pathway as a precursor to cholesterol, making it both biodegradable and biocompatible. The commercial production of squalene often uses squalene derived from shark livers [13,14,15,16]. The development of these squalene-based adjuvants has allowed for the creation of effective vaccines against seasonal, avian, and pandemic influenzas. These vaccines have been approved in Europe and in the United States for use in adults, children, infants, the elderly, and even pregnant women [17,18,19]. The general concept of emulsion-based adjuvants can be traced back to 1930s Freund’s adjuvant which is as old as the alum. Unfortunately, Freund’s adjuvant causes severe reactions and is too toxic for humans [20,21]. The squalene-based emulsions are superior to Freund’s adjuvant and aluminum salts in terms of their safety and adjuvant activities. Nevertheless, it also uses an empirical approach by formulating compounds for increasing vaccine immunogenicity without a clear understanding of the underlying mechanisms. Therefore, as with aluminum salts, although there are many hypotheses as to the mechanisms of squalene-based adjuvants to exert their immunostimulatory effects, the exact molecular mechanism for the adjuvanticity of these emulsions remains unclear. Furthermore, immune responses elicited by these emulsions are relatively mild compared with some immunostimulants such as the toll-like receptor (TLR) agonists [22,23,24].

Recently, TLR agonists have been extensively studied for use as adjuvants to increase the effectiveness of vaccines [25]. Some TLR agonist-based adjuvants are currently being used in licensed vaccines. AS04, which contains a TLR4 agonist monophosphoryl lipid A (MPL), was first formulated as an adjuvant in the hepatitis B virus (HBV) vaccine and then was used in the human papillomavirus (HPV) vaccine. These AS04-adjuvanted vaccines were licensed in the early 2000s [26,27,28]. AS01 is another MLP-containing adjuvant. The AS01-adjuvanted malaria vaccine and the recombinant zoster vaccine were licensed in 2015 and 2017, respectively [29,30,31]. CpG-1018, a TLR9 agonist, was used as an adjuvant in a hepatitis B vaccine licensed in 2017 [32,33]. Since 2020, 33 vaccines have been approved for use or emergency use in at least one country for SARS-CoV-2. Twenty-three of them are adjuvanted inactivated or protein subunit vaccines [34]. Besides those formulated with alum salt, Soberana 02 is a tetanus toxoid conjugated vaccine [35]. Nuvaxoid (NVX-CoV2373) and COVOVAX are adjuvanted with Matrix-M, a Novavax formulation composed of nanoparticles from saponins, cholesterol, and phospholipids [36]. Adjuvants for Corbevax, Soberana Plus, MVC-COV1901, Covaxin, and SpikoGen contain TLR agonists (Figure 1). In this paper, we comprehensively review the functional mechanisms of TLR agonist-based adjuvants and the recent progress in the development of TLR agonists as vaccine adjuvants for infectious diseases.

## 2. Toll-Like Receptors

Research into innate immunity has made great strides since the discovery of TLRs and various pattern recognition receptors (PRRs) in the late 1990s and early 2000s. This research has led to the understanding of various molecular and cellular mechanisms for the initiation and development of immune responses to protect humans from microbial infections. Based on their ligand recognition and structural features, PRRs can be divided into several major groups—TLRs, C-type lectin receptors, the nucleotide-binding oligomerization domain (NOD)-like receptors (NLR), retinoic acid-inducible gene I (RIG-I)-like receptors (RLR), and absent in melanoma-2 (AIM2)-like receptors (ALRs) [37,38,39]. Of them, TLRs are the most well characterized PRRs. Compared with the other PRRs, TLRs have a broader ligand recognition profile. The structural features of TLR ligands are made up of lipids, polysaccharides, proteins, and nucleic acids. In all, 13 TLRs (TLR1-TLR13) have been identified in mammals. Of these 13, TLRs 1–10 are expressed in humans. These TLRs are type I transmembrane proteins with structural features including an extracellular domain, a transmembrane region, and a cytoplasmic Toll/IL-1 receptor (TIR) domain [40,41,42,43,44]. TLR agonists bind to extracellular domains, and the TIR domain interacts with downstream signaling molecules to initiate cell signaling. The TLRs have different cellular locations, and they are able to recognize a wide variety of microbes’ pathogen-associated molecular patterns (PAMPs) [45,46,47].

TLRs 1, 2, 4, 5, 6, and 10 are expressed on the cell surface to sense microbial pathogens [45,46]. TLR2 recognizes a broad range of PAMPs including peptidoglycan, lipoteichoic acids, lipoproteins, lipoarabinomannan, glycophosphatidylinositol anchors, porins, and zymosan [48,49,50,51,52]. This TLR can form heterodimers with TLR1 or TLR6 to differentially interact with different lipoproteins. The TLR2-TLR6 complex preferentially recognizes mycoplasma macrophage-activating lipopeptide 2, whereas the TLR2-TLR1 heterodimer more specifically recognizes bacterial lipoproteins and triacyl lipopeptides [53,54]. TLR4 was the first mammalian TLR to be identified as being involved mainly in recognizing lipopolysaccharides (LPS) on the outer membrane of Gram-negative bacteria [55]. TLR5 recognizes flagellin, which is a component of bacterial flagella [56]. TLR10 was last human TLR to be identified and characterized. It is predominantly expressed on cell surfaces where it may detect some TLR2 ligands. There have also been reports that this TLR is expressed in endosomes and recognizes double stranded (ds)RNA. In addition, TLR10 may detect influenza viruses by sensing viral RNA-protein complexes [57,58]. In contrast, TLR3, TLR7, TLR8, and TLR9 are localized in intracellular vesicles including endosomes [45,46]. TLR3 recognizes dsRNA, which is generated during viral replication within infected cells [59]. TLR7-9 comprises a TLR subfamily with members containing longer extracellular domains [42,43]. TLR7 and TLR8 recognize single-stranded (ss)RNA viruses, such as the vesicular stomatitis virus or the influenza virus, and can be activated by certain synthetic chemical compounds, such as imiquimod, loxoribine, and resiquimod [60,61]. TLR9 is essential for the response to microbial unmethylated CpG DNA and is activated by synthetic CpG-oligodeoxynucleotides (CpG-ODNs), which are ODNs containing CpG-dideoxynucleotides and a phosphorothioated backbone [62,63,64]. The synthetic TLR agonists are developed by mimicking the molecular patterns and the immunostimulatory activities of their natural ligands and investigated for various therapeutic applications. However, the expression pattern of a TLR is cell-type and activation status dependent; there are also prominent differences in cell expression patterns of a TLR between mice and humans. These make direct translation rather complicated [65,66,67,68,69].

## 3. Signal Transductions for the Regulation of Gene Expression following TLR Activation

**Figure 2:**
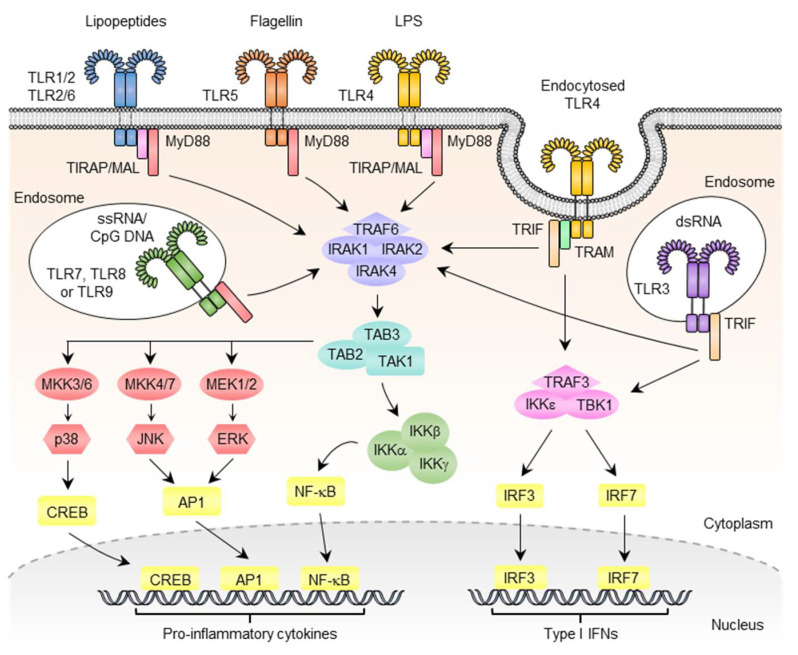
Overview of TLR signaling pathways. TLRs localize to the cell surface and in endosome compartments, where they detect pathogens from microbes. Upon stimulation, TLRs activate two major downstream adaptor proteins, MyD88 and TRIF. Engagement of the signaling adaptor molecules stimulates downstream signaling cascades that involve the production of proinflammatory cytokines and interferons (IFNs). PAMP, pathogen-associated molecular pattern; DAMP, damage-associated molecular pattern; LPS, lipopolysaccharide; MyD88, myeloid differentiation primary response 88; MAL, MyD88 adaptor-like protein; TRIF, TIR domain-containing adaptor-inducing interferon-β; TRAM, TRIF-related adaptor molecule; TRAF, tumor necrosis factor receptor-associated factor; IRAK, interleukin-1 receptor-associated kinase; TBK1, TANK-binding kinase 1; TAB, TAK1-binding protein; TAK1, TGF-β-activated kinase 1; IKK, inhibitor of NF-κB kinase; NF-κB, nuclear factor kappa-light-chain-enhancer of activated B cells; ERK, extracellular signal-regulated kinase; JNK, c-Jun N-terminal kinase; AP-1, activator protein 1; CREB, cAMP-responsive element-binding protein; IFN, interferon; IRF, IFN regulatory factor.

TLR monomers form heterodimers or homodimers for ligand binding. The TLR dimer then recruits TIR domain-containing adaptor proteins to initiate downstream signal transductions. These adaptor proteins include myeloid differentiation primary response 88 (MyD88), TIR-domain-containing adapter-inducing interferon-β (TRIF)/toll-like receptor adaptor molecule 1(TICAM-1), toll-interleukin 1 receptor (TIR) domain-containing adapter protein (TIRAP)/MyD88-adapter-like (Mal), TRIF-related adaptor molecule (TRAM)/Toll/interleukin-1 receptor domain-containing protein (TIRP), sterile alpha and armadillo motif-containing protein (SARM), and B-cell adapter for PI3K (BCAP). In addition, SLP adaptor and CSK interacting membrane protein (SCIMP), a non-TIR transmembrane protein, was identified as a proximal TLR adaptor protein [70,71]. Signal transductions initiated by TLR activation can be classified into MyD88-dependent and MyD88-independent pathways (Figure 2). All TLRs except TLR3 utilize MyD88-dependent pathways to activate transcription factors, including nuclear factor kappa-light-chain-enhancer of activated B cells (NF-κB) and activator protein 1 (AP-1) for the production of proinflammatory cytokines such as interleukin (IL)-1, IL-6, tumor necrosis factor (TNF)-α, and chemokines. In this pathway, MyD88 recruits IL-1R-related kinase 4 (IRAK4) and activates IRAK1 and IRAK2 to form a complex with TNF receptor-associated factor 6 (TRAF6), which then promotes the association of transforming growth factor (TGF)-β-activated kinase 1 (TAK1) with TAK-binding protein (TAB)2 and TAB3. This TAK1/TAB2/TAB3 complex activates an IκB kinase (IKK) complex, which, in turn, phosphorylates and promotes the ubiquitous degradation of IκB and the translocation of NF-κB into the nucleus for transcription of the NF-κB-controlled genes. In addition, in this pathway, TARF6 activates mitogen-activated protein kinases (MAPKs) including the c-Jun N-terminal kinase (JNK) and p38 MAPK for activation of AP-1-regulated genes [72,73]. Distinct from other TLRs, TLR3 and TLR4 utilize the MyD88-independent pathway for signal transductions (Figure 2). In this pathway, TLR activation recruits TRIF to activate interferon regulatory factors (IRFs), NF-κB, and AP-1 for the production of type I interferons (IFNs) and proinflammatory cytokines. IRF3/7 activation involves a TBK1-IKKε/IKKi complex; NF-κB and AP-1 activation involves TRAF6 and RIP [74,75,76]. In general, MAL bridges MyD88 to the ligand-activated cell surface of TLRs [77,78], and TRAM binds to the TIR domain of TLR4 for recruitment of TRIF to TLR4 in the endosome [74,79]. The two other TIR domain-containing adaptor proteins, SARM and BCAP, were reported to have negative regulatory functions on TLR signaling [80,81,82,83]. In contrast, SCIMP acts as a scaffold protein to mediate phosphorylation of tyrosine residues on TLRs, which then propagate downstream signal transductions for the production of inflammatory cytokines [84,85]. In addition to the utilization of adaptor proteins, cellular location also determines the consequences of TLR activation. For example, TLR4 activation on the cell surface and in endosomes results in the utilization of distinct cellular signaling for the differential production of inflammatory cytokines and type I IFNs. The engagement of TLR9 by its ligand in distinct endosomal compartments of plasmacytoid dendritic cells (pDCs) can result in the differential activation of proinflammatory cytokines and the production of type I IFNs [86,87,88,89].

## 4. Adjuvant Effects Elicited by TLR Activation

**Figure 3:**
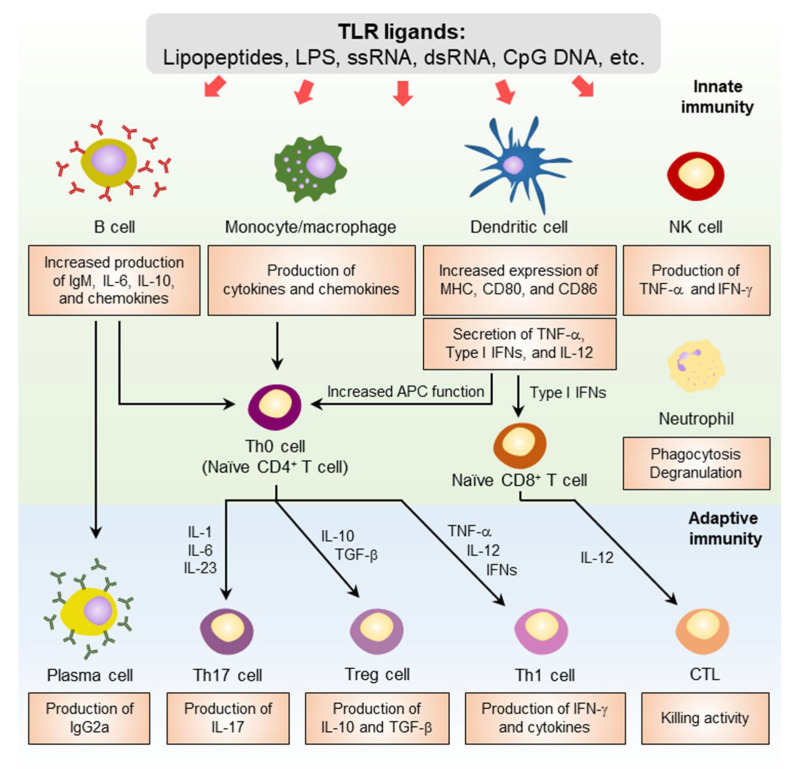
Immune responses elicited by TLR activations. TLR ligands directly activate APCs, such as monocytes/macrophages, DCs, and B cells. The surface expression of maturation markers and costimulatory molecules, including CD80, CD86, and MHC-II are increased in DCs and macrophages. The production of proinflammatory cytokines by activated APCs such as IL-1, IL-12, and type I IFNs promote naïve CD4^+^ T-cell differentiation to Th1, Th17, and Treg subsets. NK cells and CTLs are also activated, secreting IFN-γ and increasing killing activity, respectively. Moreover, B-cell activation by TLR ligands increases the production of IgM, IL-6, IL-10, and chemokines. Then the B cells differentiate to plasma cells, which increases their antigen-specific IgG production. APC, antigen-presenting cells; DC, dendritic cell; CTL, cytotoxic T lymphocyte; MHC, major histocompatibility complex; TNF, tumor necrosis factor; IL, interleukin.

Activation of TLRs by their ligands in cells triggers innate and adaptive immune responses (Figure 3) [90,91]. Within a few hours of TLR stimulation, innate immune responses are initiated. In the context of TLR agonist-adjuvanted vaccines, this early phase of the adjuvant-elicited antigen-independent innate immune response is crucial for the subsequent development of an effective antigen-specific immune responses. During this phase, gene expression increases; chemokines and proinflammatory cytokines are released from TLR-expressing cells; and innate immune cells including monocytes, macrophages, DCs, natural killer (NK) cells, and neutrophils are recruited to the vaccine injection site. In antigen-presenting cells (APCs), such as DCs, the expression of cell surface costimulatory molecules, including the cluster of differentiation 80 (CD80), CD86, and molecules of the major histocompatibility complex (MHC) are increased. The APCs at the injection sites uptake the vaccine antigen and migrate to the draining lymph node (LN) or to prime lymphocytes [92,93]. These TLR agonist-activated early immune responses are followed by a second phase of adaptive immune responses that occur several days later. During this second phase, activated APCs produce cytokines to shape the differentiation of naïve CD4^+^ T cells into different T helper (Th) cell subsets. TNF-α, IL-12, and IFNs promote Th1 polarization and IL-1, IL-6, and IL-23 promote Th17 polarization. Th1 cells produce IFN-γ and proinflammatory cytokines, and Th17 cells are the major source of IL-17. The second phase of adaptive immune responses results in the expansion of antigen-specific CD8^+^ T cells. B cells play an essential role in the development of antibody response to infection following vaccination. TLRs regulate the development and differentiation of B cells and increase the production of antigen-specific antibodies. In combination with B-cell receptor stimulation by antigen and CD40 stimulation by follicular Th cells, TLR activation initiates activation of antigen-specific follicular B cells, which then developed into germinal center (GC) B cells. In this stage, TLR activation enhances GC responses including proliferation, somatic hypermutation, and class switch recombination in the B cells. These cells can further differentiate into antibody-secreting plasma cells or memory B cells, which are long-lived B cell populations. Further, TLR activation in plasma cells enhances antibody production [94,95,96,97].

Despite the fact that different TLR ligands share a common mode of action as shown in Figure 2, their immunological inducing profiles are not entirely the same. The adjuvant activities of the TLR4 agonist (MLP), TLR7/8 agonist (R848), and TLR9 agonist (CpG-2006) were compared in rhesus macaques (*Macaca mulatta*). An intradermal injection of these primates with TLR agonists induced a rapid expansion of neutrophils and CD14^+^ monocytes in their blood. Results of the primate injections, however, revealed that the TLR7/8 and TLR9 ligands preferentially induced the activation of myeloid dendritic cells (mDCs) and plasmacytoid dendritic cells (pDCs) and the production of IFN-γ-inducible protein 10 (IP-10) and type I IFNs [98]. Studies involving human blood also revealed the differences in the cytokine-inducing profiles of the TLR4, TLR7/8, and TLR9 agonists [98,99]. The fact that different TLR ligands have some shared but also distinct adjuvant effects is understandable since these TLRs have overlapping but different cell-type expression profiles [45,46]. Additionally, these TLR ligands preferentially utilize different signal transductions and transcription factors for controlling gene expression (Figure 2).

The adjuvant activities of the TLR ligands and TLR-independent adjuvants were also compared. In one study, CpG-ODN, MF59, and alum were shown to have distinct gene regulation profiles, yet they nevertheless modulated a common set of genes and promoted antigen-presenting cell recruitment at the site of intramuscular (i.m.) injection in mice [100]. In another study, the immune stimulatory effects of TLR-dependent (Pam3CSK4, R848, CpG-ODN) and TLR-independent (MF59 and alum) adjuvants were compared. In contrast to the TLR agonists, MF59 and alum did not modulate the transcription of interferon-related genes in mouse splenocytes following in vitro stimulation [101]. After i.m. injection, R848 and CpG-ODN were shown to strongly regulate interferon-related genes in muscles. In addition, R848 affected regions distant from the injection site and regulated gene expression in the draining LN leading to the activation of polyclonal T cells and B cells [102,103]. Further, omics study revealed that vaccine formulated with the Env human immunodeficiency virus (HIV) antigen with alum and either the TLR4 agonist or the TLR7 agonist activated distinct immune responses in nonhuman primates. The TLR4 agonist upregulated the expression of inflammatory genes. In contrast, the TLR7 agonist preferentially activated antiviral and IFN genes [104].

## 5. TLR Agonist-Based Adjuvants Formulated in Licensed Vaccines

Because TLR agonists possess potent immunostimulatory activities and have a distinct model of action from traditionally used alum and emulsion-based adjuvants, they were investigated in the hope that they could be developed as adjuvants for increasing the efficacy of vaccines. So far, MPL, a TLR4 agonist, and CpG-1018, a TLR9 agonist, have been used in licensed vaccines to enhance the immune response to the microbial infections.

### 5.1. MPL

LPS is the major constituent of the outer membrane of all Gram-negative bacteria and is recognized as being involved in the septic shock elicited by Gram-negative bacteria [105,106]. Lipid A is a moiety of LPS that plays a key role in LPS activation of TLR4-mediated immune responses. Bacterially derived LPS and lipid A usually are too toxic for therapeutic applications. MPL is a detoxified form of LPS from *Salmonella minnesota* (*S. minnesota*). The toxicity of MPL is about 1000-fold less than *S. minnesota* LPS [107,108]. The adjuvant AS04 is formulated with alum and MPL. An AS04-adjuvanted HBV vaccine (Fendrix) was first licensed in 2005, and an HPV vaccine (Cervarix) was licensed in 2007 by the European Medicines Agency (EMA) [27,28,109,110]. Both the AS04-adjuvanted HPV and HBV vaccines induce a higher level of antibodies than the same vaccine adjuvanted with just alum, demonstrating the added value of the TLR4 agonist in the vaccine [111,112]. The development of AS04-adjuvanted HPV vaccines were intended for the mass immunization of girls and young women to prevent cervical disease associated with certain oncogenic HPV types. AS04 induced a sustained immune response in the HPV vaccines compared with the vaccine only adjuvanted with alum [113]. TLR4 activation by MPL or AS04 leads to a rapid production of cytokines and chemokines with an infiltration of higher numbers of immune cells into the injected muscle and draining LN within three to six hours. AS04 promotes a greater uptake of antigens by APCs compared with alum, leading to the activation of antigen-specific T cells and B cells and persistent antibody and cellular immune responses. Furthermore, the AS04-adjuvanted HPV vaccine induces a higher level of IFN-γ, which is a key cytokine of the Th1-biased response, compared with the alum-adjuvanted vaccine, indicating that AS04 is more effective in inducing a Th1 immune response. Alum does not appear to synergize with MPL in the immune stimulations, but the presence of alum prolongs the cytokine response at the injection site likely due to a depot effect [114,115]. The AS04-adjuvanted HBV and HPV vaccines showed acceptable clinical reactogenicity and safety profiles. Local transient inflammatory responses occurred at the injection site in a few hours to a few days after vaccination and resolved within several days. The incidence of serious adverse events and potential immune-mediated disease (pIMD) were similar in the recipients of the AS04-adjuvanted vaccines and in the recipients of the control. Furthermore, data from clinical trials and postmarketing evaluations showed no evidence of increased risk of adverse pregnancy outcomes or development of autoimmune events in individuals vaccinated with AS04-adjuvanted vaccines [116,117,118].

AS01 and AS02 are two other MPL-containing adjuvants. They are distinct from AS04 because they are formulated by combining two immune stimulants: MPL and QS-21. The QS-21 is purified from the bark extract of *Quillaja saponaria* Molina (fraction 21), which contains water-soluble triterpene glycosides, also referred to as saponins. AS01 is formulated in cholesterol-containing liposomes. In contrast, AS02 is formulated in an oil-in-water emulsion [29,30,119,120]. While the functional mechanism of MPL is better defined, some molecular mechanisms for the immunostimulatory activities of QS-21 have only recently been proposed. QS-21 has been shown to activate caspase-1, which promotes the maturation and production of IL-1β and IL-18. The activation of caspase-1 is NOD-, LRR-, and pyrin domain-containing protein 3 (NLRP3) inflammasome-dependent in vitro, although NLRP3 does not seem to play a role in adjuvanticity in vivo. QS-21 has also been shown to activate Syk kinase following cholesterol-dependent endocytosis and lysosomal destabilization, similar to the functional mechanisms of other adjuvants such as alum [121,122,123,124]. MPL and QS-21 could work synergistically to increase the release of cytokines and chemokines to enhance the recruitment of monocytes and dendritic cells. In addition, they synergistically enhance antigen-specific responses through an early induction of IFN-γ by cells in the draining LN, which, in turn, promotes a strong Th1 response and antigen-specific T-cell production [125]. AS01 has been used in two licensed vaccines. The RTS, S/AS01 (trade name Mosquirix) vaccine contains a recombinant RTS hybrid antigen and AS01_E_ (25 μg of MPL and 25 μg of QS-21). This vaccine was approved for use against malaria by the EMA in 2015 [30]. The recombinant zoster vaccine (RZV, Shingrix) vaccine contains varicella zoster virus (VZV) glycoprotein E and AS01_B_ (50 μg of MPL and 50 μg of QS-21). This vaccine for use against the herpes zoster was approved by the Food and Drug Administration (FDA) in 2017 [31,126]. AS02 was ever investigated as an adjuvant for the malaria vaccine. Nevertheless, because the AS01_E_-adjuvanted RTS, S vaccine displayed superior immunological responses, investigations into a potential RTS, S/AS02 malaria vaccine were terminated. Similarly, the development of AS02-adjuvanted HIV and tuberculosis vaccines were discontinued because of the better adjuvanticity of AS01 [127,128,129,130].

### 5.2. CpG-1018

The immunostimulatory activity of a CpG-ODN is determined by its structure, which is created by its nucleotide sequence and a backbone modification [131,132]. Based on their structures, CpG-ODNs can be divided mainly into three types. Type A CpG-ODNs contain a central phosphodiester palindrome region with a CpG-hexamer motif in the palindrome and poly (G) sequences and a phosphorothioate backbone attached to the 5′ and 3′ ends. This type of CpG-ODN activates the maturation of pDCs and induces the production of IFN-α but has little effect on B-cell activation. Type B CpG-ODNs contain a phosphorothiolate backbone throughout their entire sequence with one or several CpG-hexamer motifs. This type of CpG-ODN strongly induces B-cell proliferation, the production of inflammatory cytokines, and has some effect on the maturation and activation of pDCs, monocytes, and NK cells. Type C CpG-ODNs contain a phosphorothioate backbone with one or two CpG-hexamer motifs and a palindromic sequence at the 3′ end, and they have an immune stimulatory profile in between those of type A and type B CpG-ODNs [133,134,135]. In humans, the TLR9 is constitutively expressed in pDCs and B cells, and to some extent is also expressed in monocytes/macrophages, cDCs, activated neutrophils, and T cells. These cell-type effects of different forms of CpG-ODNs indicate that they have different models of action for distinct immune stimulatory activities, nevertheless, so far type B CpG-ODNs are the most commonly used CpG-ODNs in preclinical and clinical trials [136,137,138,139]. CpG-1018 is a type B CpG-ODN. Heplisav-B, approved by the FDA in 2017, is a CpG-1018-adjuvanted hepatitis B vaccine. In phase 3 clinical studies, Heplisav-B was compared with Engerix-B, which is adjuvanted with alum salt and has been used for more than 30 years in adults. Administration with two doses of Heplisav-B induced higher seroprotective responses with a faster onset rate compared with the administration of three doses of Engerix-B. In addition, the safety profiles of Heplisav-B and Engerix-B are comparable. A more recent phase 3 clinical trial investigated the efficacy and safety of these two vaccines in patients with type 2 diabetes mellitus aged 60–70 years. Two doses of Heplisav-B provided a higher level of seroprotection against HBV than three doses of Engerix-B with similar safety profiles [32,33]. So far, there is not much detailed information about the immunostimulatory profiles of CpG-1018-adjuvant vaccines in humans compared with what is known about HBV vaccines with other adjuvants. In general, CpG-ODN can activate the maturation of DCs to become professional antigen-presentation cells, induce Th1 responses to support the production of IFN-γ and CD8^+^ T cells, and accelerate antibody responses for protective immunity [140,141,142].

## 6. TLR Agonist-Based Adjuvants Employed in Clinically Investigated Vaccines and Vaccines Approved for Emergency Use

In addition to MPL and CpG-1018, other TLR agonists were investigated or are currently being investigated as adjuvants for vaccines against various infections. These are enumerated in Table 1 and discussed in the following sections.

### 6.1. TLR2 Agonist-Based Adjuvants

The most frequently used adjuvant for TLR2 activation is macrophage-activating lipopeptide-2 (MALP-2) and its synthetic analogs, dipalmitoyl-S-glycerylcysteine (Pam2Cys) and tripalmitoyl-S-glycerylcysteine (Pam3Cys). Strategies to develop TLR2 agonist-adjuvanted vaccines include formulating synthetic TLR2 agonists into vaccine adjuvants and conjugating TLR2 agonists to antigens [143,144]. Because they are easily incorporated during peptide synthesis, various TLR2 ligand-conjugated peptide vaccines were investigated in earlier studies. Lipo-4 and Lipo-6 are HIV-targeting vaccines using palmitic acid-extended peptide antigens. Their efficacies were evaluated in phase 1 and phase 2 studies in healthy and HIV-infected adults. The vaccines generated IgG antibodies and specific cytotoxic T lymphocyte (CTL) responses. Nevertheless, they failed to boost enough response to HIV-specific CTLs in HIV-infected subjects [145,146]. Theradigm-HBV is a palmitic acid-conjugated peptide vaccine for HBV infection that was tested in phase 1 and phase 2 studies in healthy and HBV-infected adults. The conjugation with palmitic acid significantly increased the responses of helper T lymphocytes and CTLs to the peptide antigens compared with the unconjugated vaccine. The vaccine increased the response of HBV-specific CTLs and persisted for more than nine months in healthy adults. Nevertheless, the CTL responses were not induced in patients with HBV [147,148]. More recently, TLR2 agonists including VLA-15 and XS15 have been investigated. VLA-15 is a multivalent recombinant vaccine candidate that targets six serotypes of *Borrelia*. It targets the outer surface protein A (OspA) of lyme disease spirochete, *Borrelia burgdorferi* [149,150]. OspA is a lipoprotein that activates TLR1/2. Mice genetically deficient in either TLR2 or TLR1 produced low titers of antibodies after OspA immunization [151], thus making it a type of TLR2 agonist-linked antigen. VLA-15 is currently in phase 2 clinical studies for Lyme disease. XS15 is a water-soluble synthetic Pam3Cys-derivative TLR1/2 activator. It was originally designed for use as adjuvant in cancer vaccines. It induced strong responses of Th1 CD4 and CD8 T cells in human volunteers with a single injection of XS15 mixed with uncoupled peptides in a Montanide ISA 51 water-in-oil emulsion [152]. CoVac-1 is a XS15-adjuvanted peptide vaccine composed of SARS-CoV-2 T-cell epitopes derived from various viral proteins for inducing T-cell immunity to combat COVID-19. In the phase 1 study, CoVac-1 showed an acceptable safety profile and induced potent T-cell-mediated responses, which supports the evaluation of this vaccine in a phase 2 trial for patients with antibody production deficiencies [153,154,155].

### 6.2. TLR3 Agonist-Based Adjuvants

TLR3 recognizes dsRNA. Polyriboinosinic:polyribocytidylic acid (I:C) is structurally similar to dsRNA and is an archetypal synthetic TLR3 agonist. This TLR3 agonist caused major safety problems in phase 1 and phase 2 clinical trials of patients with cancer [156]. Nevertheless, poly I:C activates retinoic acid-inducible gene I (RIG-I) and melanoma differentiation-associated protein 5 (MDA5) in addition to TLR3, therefore, its immunostimulation cannot solely be attributed to TLR3 activation [157,158]. Other agonists developed for TLR3 activation include poly I:C_12_U (Ampligen, Rintatolimod), poly-ICLC (Hiltonol), and PIKA. Poly I:C_12_U was modified from the poly I:C molecule by adding mismatched uracil and guanine bases at specific intervals along the RNA chain. Poly I:C_12_U stimulated IFN production like poly I:C, yet unlike poly I:C, it signals solely through the TLR3 receptor and not through MDA-5 and with much lower toxicity. Poly I:C_12_U was investigated in earlier clinical studies as an adjuvant for nasal influenza vaccine, a therapeutic agent against HIV, and as an adjuvant for cancer vaccines. In addition, it was investigated for the treatment of chronic fatigue syndrome in phase 2 and phase 3 studies. This TLR3 agonist is safe for use for inducing immune responses including the responses of antigen-specific CTLs [159,160,161,162,163]. Poly-ICLC is a poly-L-lysine carboxymethylcellulose stabilized poly I:C. Administration of poly-ICLC to patients with anaplastic astrocytomas and glioblastomas was implicated in prolonged survival. In a phase 1 study, this TLR3 activator markedly increased the immunogenicity of a peptide vaccine in patients with ovarian cancer. In another study with patients with breast cancer treated with the poly-ICLC-adjuvanted peptide vaccine, the poly-ICLC setting was safe and provided adjuvant activity [164,165,166]. Although most of the clinical investigations of poly-ICLC involved investigating its function in cancer immunotherapy and as an adjuvant for cancer vaccines, it is currently being investigated for its safety in the nasal delivery of healthy COVID-19-vaccinated subjects [167]. It also has been investigated in phase 1 clinical trials as an adjuvant for an anti-HIV vaccine containing a fusion protein with a human monoclonal antibody specific for the dendritic cell receptor, DEC-205 (CD205) conjugated to the HIV gag p24 protein [168]. PIKA is a kanamycin- and calcium-stabilized poly I:C. It was investigated in phase 1 and phase 2 clinical trials as an adjuvant for a rabies vaccine comprising inactivated rabies virus (PIKA rabies vaccine). The PIKA-adjuvanted vaccine was safe, well tolerated, and more immunogenic than the commercially available vaccine in healthy adults [169,170]. In another study for a PIKA-adjuvanted COVID-19 vaccine, the recombinant S trimeric protein-containing vaccine induced high levels of neutralization titers and protected nonhuman primates from virus replication in the lungs following a SARS-CoV-2 challenge [171]. Currently, this PIKA COVID-19 vaccine is being investigated in a phase 1 clinical trial [172].

### 6.3. TLR4 Agonist-Based Adjuvants

In addition to their use in licensed vaccines against HPV, HBV, malaria, and herpes zoster, MPL-containing adjuvants have been employed in clinically investigated vaccines for tuberculosis, *Clostridium difficile*, HIV, herpes simplex virus, Norwalk virus, and respiratory syncytial virus (RSV) [173,174,175]. Moreover, because of the diseases’ characteristics, the two AS01-adjuvanted malaria and herpes zoster vaccines have been further investigated in different populations with subjects of different ages. Malaria is a parasitic disease transmitted by a mosquito’s bite. The major populations targeted for malaria vaccines are infants and children in the low-to-middle income countries of Africa and Southeast Asia. Current existing data revealed that the RTS, S/AS01 vaccine is safe, well tolerated, and immunogenic in children. Although it prevents malaria, its efficacy is modest, showing about a 26% efficacy in infants and a 36% efficacy in children in the four-year follow-up [176,177]. In contrast, herpes zoster is caused by reactivation of the latent varicella zoster virus from a previous infection, which is more likely to occur in people with age-related declines in immunity or in immune-suppressed populations. RZV has shown a remarkable efficacy of >90% in preventing herpes zoster in the elderly [178,179]. In addition, results of a recent phase 3 clinical study showed that RZV was immunogenic in chronically immunosuppressed renal transplant recipients, and the unsolicited adverse events, serious adverse events, and potential immune-mediated diseases (pIMDs) were similar between the RZV vaccination group and the placebo group [180].

MPL is processed from an extract mixture of Salmonella R595 lipid A, which is not favorable for controlling the quality of the product. Therefore, synthetic lipid A analogs for TLR4 activation, such as glucopyranosyl lipid A (GLA), were developed. GLA is formulated as an aqueous nanosuspension (GLA-AF), oil-in-water emulsion (GLA-SE), or liposome-QS-21 (GLA-LSQ) in vaccines for various infectious diseases [181,182,183]. Early stage clinical studies of GLA-AF-adjuvanted vaccines for hookworm, *Schistosoma mansoni*, and HIV revealed that these vaccines were safe, well tolerated, and induced an antibody response [184,185,186]. GLA-SE-adjuvanted vaccines for influenza, RSV, malaria, and tuberculosis were evaluated in phase 1/2 studies. In general, these vaccines did not raise safety concerns, and they induced antibody and cell-mediated immune responses [187,188,189,190]. In a phase 2 study for the comparative investigation of alhydrogel-adjuvanted and GLA-SE-adjuvanted H5N1 influenza vaccines, the GLA-SE-adjuvanted vaccine induced higher hemagglutination-inhibition responses than the alhydrogel-adjuvanted vaccine. In addition, the GLA-SE-adjuvanted vaccine was able to elicit both a humoral response and sustained cell-mediated immunity in healthy adults, suggesting the efficacy of this GLA-SE adjuvant [187]. A malaria subunit vaccine composed of GLA-LSQ and plasmodium falciparum circumsporozoite protein (CSP) is being investigated in a phase 1 clinical study. Interim results have shown favorable safety and immunostimulatory characteristics for this vaccine. The adjuvanted vaccine achieved a >90-fold rise in the geometric mean anti-CSP IgG antibody titer at 28 days after three doses [189].

RC-529 is another analog of lipid A distinct from MPL and GLA in that its disaccharide backbone is replaced by a monosaccharide backbone [181]. In a randomized trial, a RC-529-adjuvanted hepatitis B vaccine (AgB/RC-210-04) was compared with an aluminum-adjuvanted hepatitis B vaccine (AgB), which contained recombinant hepatitis B surface antigens (HBsAg) and alum. The rates of seroprotection and the geometric mean anti-HBs titers were significantly higher for the AgB/RC-210-04 group. There were more local reactions in the RC-529-adjuvanted vaccine group, but the reactions were transient, and this vaccine was well tolerated [191]. A RC-529-adjuvanted hepatitis B vaccine (Supervax) was licensed in Argentina in 2003 [192].

### 6.4. TLR5 Agonist-Based Adjuvants

The TLR5 ligand, flagellin, is a major component of bacterial flagella. Because of its protein nature, it can be easily fused to peptide or protein antigens for codelivery to APCs to induce an enhanced antigen-specific response. Several vaccines with this kind of design have been evaluated in clinical trials. The plague is an infectious disease caused by *Yersinia pestis*. A recombinant protein generated by fusing the F1 and V proteins of *Y. pestis* to a hypervariable region of flagellin was designed as a vaccine for protection against the plague. In a phase 1 study, the Flagellin/F1/V vaccine induced T cells and specific antibody responses and was well tolerated. The results of this trial also suggested higher doses of the vaccine could be tested [193,194]. The ectodomain of matrix protein 2 (M2e) is highly conserved in human epidemic influenza A virus strains, and antibodies against M2e are protective in animal models. Therefore, M2e was considered as an antigen for a universal influenza virus vaccine [195,196,197]. VAX102 (STF2.4xM2e) is a recombinant fusion protein generated by linking four tandem copies of the M2e to *Salmonella typhimurium* flagellin type 2 (STF2). In phase 1/2 studies, VAX102 was safe, well tolerated, and induced high antibody levels to M2e [198]. VAX125 is an *E. coli*-expressed recombinant protein consisting of the globular head of the HA1 domain of the A/Solomon Islands/3/2006 (H1N1) influenza virus fused to the STF2. Phase 1/2 trials indicated that the VAX125 was well tolerated and generated hemagglutination-inhibition (HAI) antibodies in healthy adults and the elderly [199,200]. VAX128 is series of vaccines designed by fusing different regions of flagellin to the HA1 domain of the influenza A/California/07/2009 (H1N1) virus. Flagellin has four domains: D0-D3. The HA1 domain was fused to the C-terminal end of flagellin to generate VAX128A. In VAX128B, the D3 domain of flagellin was deleted and replaced with the HA1 domain. VAX128C was constructed by incorporating a copy of HA1 to the C-terminal end and another copy to replace D3 domain of flagellin as it was done in the creation of VAX128B [201]. The safety and immunogenicity of these designs were tested in a phase 1 study. The immunogenicities of VAX128B and VAX128C were comparable with that of VAX128A, but they were better tolerated at higher doses than VAX128A [201]. VAX128C continues to be investigated. VAX2012Q is a quadrivalent influenza vaccine composed of four hemagglutinin subunit flagellin-fused proteins. The four subunits are VAX128C (H1N1), VAX181 (H3N2), VAX173 (B-YAM), and VAX172 (B-VIC). The safety and immunogenicity at different doses of this vaccine were evaluated in phase 1/2 trials. In general, VAX2012Q elicited immune responses at all doses with no significant safety concerns [202].

### 6.5. TLR7/8 Agonist-Based Adjuvants

TLR7 and TLR8 are the most phylogenetically related of the 10 human TLRs [42]. Consequently, their recognition of ligands overlap. These two TLRs are activated by certain purine-rich ssRNAs found in viruses, and their activation can be mimicked with synthetic chemical compounds. Imidazoquinoline compounds such as resiquimod (R-848) and CL097 activate both TLRs, whereas imiquimod (R-837) predominantly activates TLR7. Other synthetic activators include CL075, a thiazoquinoline compound that activates both TLRs; loxoribine, a guanosine analog that mainly activates TLR7; and VTX-2337, a benzoazepine compound that preferentially activates TLR8 [203,204,205,206,207]. Human TLR7 is primarily expressed in pDCs and to some extent in monocytes/macrophages, T cells, and B cells. Human TLR8 is predominantly expressed in myeloid DCs and also is expressed in monocytes/macrophages and T cells [69]. The expression of these two TLRs in DC subsets is an important reason for the TLR7/8 agonist to be considered as a vaccine adjuvant. A 5% imiquimod cream (trade name Aldara) was licensed for treatment of HPV-mediated external genital warts, actinic keratosis, and superficial basal cell carcinoma [207]. Imiquimod and resquimod were clinically investigated as topical skin adjuvants to enhance the intradermally injected influenza and HBV vaccines. Topical treatment of imiquimod before intradermal injection of the influenza vaccine significantly improved the vaccine response in young, healthy individuals and the elderly with chronic illnesses. In contrast, the adjuvant effect of this treatment was not seen in immunocompromised patients. Topical treatment with imiquimod was well tolerated, and adverse effects were mild, transient, and primarily seen in localized reactions [208,209,210]. Nevertheless, due to their small size, imidazoquinolines have an unfavorable pharmacokinetic property of rapidly diffusing from the injection site and cause systemic immune activation rather than localized stimulation. Severe side effects were reported for oral or systemic use of imiquimod and resiquimod [211,212]. Thus, other strategies for using TLR7/8 agonists as adjuvants have been investigated.

3M-052 is an imidazoquinoline compound structurally similar to resiquimod. This TLR7/8 agonist has an 18-C fatty acyl chain that confers this compound an enhanced hydrophobicity for improved bioavailability at the immunization site and reduced systemic dissemination [213]. In addition, this lipidation enables 3M-052 to be more amenable to incorporation into lipid-based formulations such as emulsions or liposomes. HIV vaccines formulated with alum-3M-052 or 3M-052 encapsulated in PLGA nanoparticles have been shown to induce increased immune and antibody responses and provide protection against HIV challenges in macaques. The alum-3M-052-adjuvanted HIV vaccine has been investigated in phase 1 clinical trials for humans [214,215,216]. In addition to this, an alum-absorbed imidazoquinoline (Algel-IMDG) was formulated in a whole virion inactivated SARS-CoV-2 vaccine (COVAXIN, BBV152). The interim results of a phase 3 trial in India showed that this vaccine was well tolerated with an overall estimated vaccine efficacy of 77.8%. COVAXIN has been approved by the World Health Organization (WHO) for emergency use [217,218].

Other than imidazoquinolines, an RNA-based TLR7/8 agonist CV8102 was investigated for use as a vaccine adjuvant. CV8102 activates the retinoic acid-inducible gene I (RIG-I) pathway in addition to TLR7/8. RIG-I is a cytosolic receptor that recognizes short viral dsRNA with a 5′-triphosphate. CV8102 is a polyU repeat containing ssRNA with a 5′-triphosphate modification and complexed with a small arginine-rich disulfide-crosslinked cationic peptide. CV8102 was administered alone or mixed with fractional doses of a licensed rabies vaccine in a phase 1 human trial to investigate its safety, tolerability, and immunogenicity. The results showed that this novel type of TLR7/8/RIG-I agonist was safe and significantly enhanced the immunogenicity of the rabies vaccine [219,220].

### 6.6. TLR9 Agonist-Based Adjuvants

In addition to the licensed Heplisav-B vaccine, several CpG-1018-adjuvanted vaccines are currently being investigated in clinical studies. In a phase 1 study, an HIV-1 BG505 SOSIP.664 gp140 antigen formulated with CpG-1018/alum was compared with an HIV-1 BG505 SOSIP.664 gp140 antigen formulated with TLR4 and TLR7/8 agonists in the presence or absence of alum to evaluate their safety and immunogenicity. In addition, several CpG-1018-adjuvanted COVID-19 vaccines were investigated. VLA2001 is a CpG1018/alum whole virus inactivated COVID-19 vaccine. This vaccine was well tolerated and produced both humoral and cellular immune responses in phase 1/2 studies [221]. In October 2021, VLA2001 was announced to have a geometric mean titer (GMT) for the neutralization antibodies of 803.5 compared with AZD1222′s GMT of 576.6 in a phase 3 trial (the AZD1222 is an Oxford-AstraZeneca adenoviral vector-based COVID-19 vaccine) [222]. The SCB-2019 vaccine contains an S-trimer protein formulated with a CpG-1018/alum adjuvant. Phase 1 studies revealed that this vaccine elicited robust immune responses and neutralizing activity against SARS-CoV-2 [223,224]. In September 2021, SCB-2019 announced phase 2/3 results showing 100% efficacy against severe COVID-19 hospitalization, 84% efficacy against moderate-to-severe COVID-19 cases, and 67% efficacy against all cases caused by any strain of SARS-CoV-2 [225]. Similar to SCB-2019, MVC-COV1901 is a CpG-1018/alum-adjuvanted subunit vaccine. Phase 1 and 2 studies showed that MVC-COV1901 has a good safety profile. At 28 days after the second dose of this vaccine, the GMT was 662.3 to wild-type SARS-CoV-2. This vaccine has obtained EUA approval in Taiwan [226,227,228].

In addition to CpG-1018, other CpG-ODNs have been investigated as vaccine adjuvants. CpG-7909 (also known as CpG-2006, PF-3512676, VaxImmune, ProMuneT, and Agatolimod) is the most thoroughly investigated CpG-ODN for its various immune stimulatory activities and its adjuvant activity in different vaccine candidates. When formulated with alum in different vaccines with different antigens from malaria, including the apical membrane antigen 1 (AMA1), merozoite surface protein 142 (MSP142), and BSAM-2 (AMA1 + MSP142), CpG-7909 increased antibody responses to these malaria vaccines in phase 1 studies [229,230,231,232]. When coadministrated with vaccines for hepatitis B (Engerix-B), CpG-7909 enhanced antibody responses and increased the seroprotection rate in healthy and HIV-infected adults [233,234]. Phase 1 and 2 studies revealed that AVA7909 formulated with CpG-7909 and an approved anthrax vaccine BioThrax(^®^) (Anthrax Vaccine Adsorbed, AVA) were both safe and well tolerated. The immunogenicity outcomes of the AVA7909 vaccine provided the rationale for further clinical studies, and thus a phase 3 trial was conducted [235,236]. Another CpG-7909-adjuvanted vaccine is 202-COV, which is a S-protein subunit vaccine currently investigated in phase 1/2 studies for COVID-19 [237]. COVAX-19 contains Advax-SM and an antigen from the receptor-binding domain of the SARS-CoV-2 spike protein. Advax-SM is formulated by combining delta inulin polysaccharide particles (Advax™) and CpG55.2 [238,239]. Phase 2 and 3 clinical trials of COVAX-19 against COVID-19 are being conducted, and Iran has approved this vaccine for emergency use [240].

## 7. Conclusions and Perspectives

Infectious diseases are a major threat to human health and can have serious social impacts and high global burdens. The impact of infectious disease is obvious in the midst of the ongoing COVID-19 pandemic, which began in 2019. According to the WHO data, leading up to January 2022, the global spread of SARS-CoV-2 had infected 299 million people and resulted in 5.5 million deaths [241]. Yearly worldwide seasonal influenza epidemics are estimated to cause about 3–5 million severe cases of illness and about 290,000–650,000 deaths [242]. Since their development two centuries ago, vaccines have been the major weapon in the fight against infectious disease. More effective vaccines are still needed for existing diseases such as tuberculosis, malaria, AIDS, and many others. Moreover, vaccines have been proven to be important tools for limiting the spread of emerging infectious diseases in their early stages, which provides compelling rationale for the continued technological development of safer and more effective vaccines. Adjuvants are important components of vaccine, and the choice of adjuvant determines the safety and efficacy of a vaccine. Previously, only a limited number of adjuvants for vaccines were available, and we had only a limited understanding of the functional mechanisms of the available adjuvants such as alum and squalene-based adjuvants. Some naturally derived adjuvants such as QS21 have favorable immune stimulatory activities, but their chemical synthesis is not easy because of their complex structures. 

The discovery of TLRs and their function in the regulation of innate and adaptive immune responses has led to the exploration of TLR agonists as vaccine adjuvants. The structural features needed for agonists to activate each TLR is relatively clear. Thus, we can easily chemically synthesize TLR agonists, and researchers have even been able to make more sophisticated modifications to improve their safety and effectiveness for use as vaccine adjuvants. As described in this paper, this approach has been taken for the development of most of the TLR agonists. Furthermore, the signal transductions and immune responses elicited by activation of each TLR have been thoroughly investigated. With this understanding, research has allowed for the rational design of TLR-based adjuvants using a combination of agonists to different TLRs or to other PRRs for synergistic adjuvant activity. Immune stimulators that target multiple TLRs or other PRRs simultaneously have also been developed; CV8102 is an example of an immune stimulator that activates both TLR7/8 and the RIG-I pathway [219,220]. Other approaches to improve the efficacy of TLR-based adjuvants include the ligation of an antigen to the TLR agonist as seen with the TLR2 and TLR5 agonists [144,194]. Another approach for improving the efficacy of TLR-based adjuvants is the formulation of a TLR agonist to nanoparticles as seen in the development of AS01 and AS02 [130].

Messenger RNA vaccines have been successfully developed for SARS-CoV-2 since the outbreak of COVID-19. The related technologies gain much interest in vaccinology. The mRNA contains inherent immune stimulatory activity that could be detrimental by resulting in unwanted immune responses or could be highly beneficial through the generated adjuvant activity. The immune stimulatory activity of mRNA is generated by activation of RNA sensors such as the TLR3, TLR7, TLR8, RIG-I, and MDA5 in cells. This activity can be optimized by adjusting the structural features of mRNA in the vaccine, which include the 5′ cap, structurally modified nucleotides, 5′ and 3′ untranslated regions, and the poly(A) region [243,244,245]. Another strategy to tailor the immunogenicity of mRNA vaccine is co-formulating it with additional adjuvant. TLR agonists have been shown to play a role in this aspect. In one study, nucleotide-modified mRNA and MLP were co-delivered and investigated for cancer immunotherapy. In another study, antigen-expressing mRNA and CpG-ODN were co-delivered with a charged-altering releasable transporter as a COVID-19 vaccine and tested in animal studies. This vaccination generated strong and long-lasting antigen-specific Th1 and antibody responses without any observed adverse effects [246,247].

This review focused mainly on the TLR agonist adjuvanted vaccines that were licensed or have entered into different stages of clinical studies. Whereas most of these vaccines are i.m. administrated, TLR agonists are also being investigated in preclinical studies and some clinical studies as adjuvant candidates for intranasally delivered vaccines. Nasal delivered Poly-ICLC was investigated for its safety in COVID-19 vaccinated adults and Ampiligen was reported in a phase 1 study to be well-tolerated when mixed in a nasal influenza vaccine (Table 1) [163,167]. MPL was shown to drive IgA production and Th1 response in animal studies and a phase 1 study has shown the safety and efficacy of an MPL adjuvanted norovirus vaccine (Table 1) [175]. In addition to these TLR3 and TLR4 agonists, the TLR5, TLR7/8, and TLR9 agonists were investigated in animal models as nasal adjuvants. When mixed with antigen, nasal administrated flagellin, imidazoquinolines, and CpG-ODNs were shown to induce IgA production and T cell responses and protected animals against viral challenges [248,249,250,251,252,253]. Furthermore, a mixture of polyI:C and CpG-ODN was shown to be an effective and safe nasal adjuvant for a COVID-19 protein subunit vaccine in rhesus macaques since no vaccine-induced adverse effect was observed even after three doses [254]. The development of nasal vaccines receives attention because of their social and economic values. Airborne infectious diseases such as COVID-19 often cause huge impacts. Compared to the i.m. administrated vaccine, nasal vaccines are able to induce mucosal immune responses characterized by mucosal secretory IgA and resident memory T cells, in addition to the induced systemic immune responses. Mucosal secretory IgA antibodies neutralize the airborne viruses in the mucosa and resident memory T cells are vital for preventing respiratory virus infection in the airway and lung. Nevertheless, the major challenges in the development of nasal vaccines are the delivery of antigen to APC in the respiratory tract and the safety issue. Approaches to overcome these obstacles include the use of a safe and effective adjuvant and delivery system to increase the efficacy and prolong the time of antigen uptake in the respiratory tract [255,256,257,258]. In this regard, TLR agonist-based nasal adjuvants are continuously being developed.

In summary, the performance of licensed TLR agonist-adjuvanted vaccines and the recent approval of those TLR agonist-based vaccines for emergency use against COVID-19 have shown the value of TLR agonists’ adjuvant activity. TLR agonists have the potential for further development utilizing different approaches such as structural modifications, combinational use, ligations to antigens, and formulations with nanoparticles for use in next-generation vaccines in the fight against infectious disease. In addition, they potentially can be used for tailoring the immunogenicity of mRNA vaccines and developed as nasal adjuvants.

## Figures and Tables

**Table 1:** TLR adjuvants in clinical development.

TLR	Adjuvant	Condition	Sponsor	Phase	Route	References
TLR2	XS15	COVID-19	University Hospital Tuebingen	I/II	S.C.	NCT04954469
	VLA15	Lyme Borreliosis	Valneva Austria GmbH	II	I.M.	NCT03769194
TLR3	poly ICLC	COVID-19 vaccinated adult HIV Chronic HIV-1 infection	Oncovir Rockefeller University Nina Bhardwaj	I I I/II	I.N. Unknown	NCT04672291 NCT01127464
	PIKA	COVID-19 Rabipur for human	Yisheng Biopharma Yisheng Biopharma	I II	I.M. I.M.	ACTRN12621001009808 NCT02956421
	Rintatolimod	Influenza	AIM ImmunoTech	I/II	I.N.	NCT01591473
TLR4	AS01	Malaria, 3–5 years old child Malaria, 5–17 months old child Clostridium infections RSV, 60–80 years Tuberculosis Herpes Zoster Hepatitis B, ≥50 years old HIV	London School of Hygiene and Tropical Medicine GSK GSK GSK GSK GSK GSK NIAID	III IIb I I II II I I/IIa	I.M. I.M. I.M. I.M. I.M. I.M. I.M. I.M.	NCT04319380 NCT03281291 NCT04026009 NCT04090658 NCT01755598 NCT00802464 NCT03866187 NCT02915016
	AS02	HIV Tuberculosis Hepatitis B	NIAID GSK Henogen	I II III	I.M. I.M. I.M.	NCT00027365 NCT00397943 NCT00291980
	AS04	HPV-16/18, aged 15–25 years Herpes Simplex, 10–17 years, female Papillomavirus, 4–6 years old child	GSK GSK GSK	III III III	I.M. I.M. I.M.	NCT00485732 NCT00224484 NCT01627561
	GLA-AF	Schistosomiasis Influenza HIV Hookworm	NIAID IDRI Imperial College London Baylor College of Medicine	I I I I	I.M. I.D./I.M. I.M. I.M.	NCT02337855 NCT01657929 NCT01922284 NCT01261130
	GLA-LSQ	Malaria Plasmodium Falciparum	University Hospital Tuebingen NIAID	I I	I.M. I.M.	NCT02647489 NCT03589794
	GLA-SE	Tuberculosis Leprosy HIV-exposed uninfected infants Schistosomiasis Malaria Leishmaniasis Influenza RSV, 60 years or older	IDRI IDRI HIV Vaccine Trials Network Fiocruz Inserm IDRI Medicago MedImmune LLC	II I I II I I II II	I.M. I.M. I.M. I.M. I.M. I.M. I.M. I.M.	NCT02465216 NCT03302897 NCT04607408 NCT03041766 NCT02014727 NCT01484548 NCT01991561 NCT02508194
	RC-529-SE	HIV	NIAID	I	I.M.	NCT00111605
	SLA-SE	Leishmaniasis	IDRI	I	I.M.	NCT02071758
	MPL	Norovirus	LigoCyte Pharmaceuticals	I	I.N.	NCT00806962
TLR5	Flagellin	Influenza Influenza Influenza Influenza, 65–75 years old Plague	VaxInnate Corporation VaxInnate Corporation VaxInnate Corporation VaxInnate Corporation NIAID	I/II I II Ib/II I	I.M./S.C. I.M. I.M. I.M. I.M.	NCT00921947 NCT01172054 NCT02434276 NCT02247362 NCT01381744
	Salmonella Typhimurium flagellin type 2	Influenza, 65 years and older Influenza Influenza	VaxInnate Corporation VaxInnate Corporation VaxInnate Corporation	II I I	I.M. I.M. I.M.	NCT00966238 NCT00603811 NCT00730457
TLR7/8	Imiquimod	Influenza, 18–30 years old adult Influenza, ≥18 years and older with chronic illness Influenza HBV vaccine in renal failure patients Hepatitis B, cirrhotic patient who did not respond to a 1st conventional HBV vaccination	The University of Hong Kong The University of Hong Kong University of Lausanne Hospitals The University of Hong Kong Central Hospital, Nancy, France	III III II II/III II	Topical application Topical application Topical application Topical application Topical application	NCT02103023 NCT04143451 NCT02960815 NCT02621112 NCT05028322
	Imidazoquinoline	COVID-19 COVID-19, 2–18 years	Bharat Biotech Bharat Biotech	III II/III	I.M. I.M.	NCT04641481 NCT04918797
	3M-052-AF	HIV	NIAID	I	I.M.	NCT04915768
	CV8102	Rabipur for humans	CureVac AG	I	I.M.	NCT02238756
	Resiquimod	Influenza, 65–75 years HBV	University of British Columbia University of British Columbia	I I/II	Topical application Topical application	NCT01737580 NCT00175435
	Vesatolimod (GS-9620)	HIV-1 positive patients	Aelix Therapeutics	II	Oral	NCT04364035
TLR9	CpG-1018	HIV HBV COVID-19 COVID-19 COVID-19 COVID-19, 12 years and older COVID-19, 12 years and older	NIAID Dynava xMedigen Vaccine Biologics Corp. Clover Biopharmaceuticals Clover Biopharmaceuticals Valneva Austria GmbH Valneva Austria GmbH	I III II I II/III III III	I.M. I.M. I.M. I.M. I.M. I.M. I.M.	NCT04177355 NCT02117934 NCT04695652 NCT04405908 NCT04672395 NCT04864561 NCT04956224
	CpG55.2	Influenza COVOD-19 COVID-19 COVID-19	NIAID Cinnagen Cinnagen Vaxine	I II III I	I.M. I.M. I.M. I.M.	NCT03945825 NCT04944368 NCT05005559 NCT04453852
	CpG-10104	Hookworm Hookworm	Baylor College of Medicine Baylor College of Medicine	I II	I.M. I.M.	NCT02143518 NCT03172975
	CpG-7909	Malaria Malaria, 1~3 years old child Malaria Malaria Pneumococcal vaccination in HIV infected adults HBV vaccination in HIV infected or uninfected adults Influenza Anthra xAnthrax, stratified by sex and age (18–50, 66–74, and ≥75 years) Anthra xAnthra xCOVID-19 COVID-19	NIAID NIAID NIAID NIAID University of Aarhus NIAID Novartis Emergent BioSolutions Biomedical Advanced Research and Development Authority Emergent BioSolutions Emergent BioSolutions Shanghai Zerun Biotechnology Co., Ltd Shanghai Zerun Biotechnology Co., Ltd	I I I I I/II I/II I I II III II I II	I.M. I.M. I.M. I.M. I.M. I.M. I.M. I.M. I.M. I.M. I.M. I.M. I.M.	NCT00889616 NCT00740090 NCT00427167 NCT00344539 NCT00562939 NCT00100633 NCT00559975 NCT01263691 NCT03518125 NCT03877926 NCT01770743 NCT04982068 NCT04990544
	IC31	Tuberculosis	Aeras	II	I.M.	NCT03512249

Abbreviations: COVID-19, coronavirus disease 2019; HIV, human immunodeficiency virus; RSV, respiratory syncytial virus; NIAID, National Institute of Allergy and Infectious Diseases; GSK, GlaxoSmithKline; IDRI; Infectious Disease Research Institute; I.D., intradermal; I.M., intramuscular; I.N., intranasal; S.C., subcutaneous. Red fonts highlight the studies of COVID-19.

## Data Availability

No new data were created in this review. Data sharing is not applicable to this review article.
